# Effects of Low-Temperature Construction Additives (LCAs) on the Performance of Asphalt Mixtures

**DOI:** 10.3390/ma15020677

**Published:** 2022-01-17

**Authors:** Yuanyuan Li, Jianlin Feng, Anqi Chen, Fan Wu, Shaopeng Wu, Quantao Liu, Ruifang Gong

**Affiliations:** 1 School of Civil Engineering and Architecture, Wuhan Institute of Technology, Wuhan 430205, China; liyy@wit.edu.cn (Y.L.); 22004010110@stu.wit.edu.cn (J.F.); 22004010123@stu.wit.edu.cn (F.W.); 2 Nottingham Transportation Engineering Centre, School of Civil Engineering, University of Nottingham, University Park, Nottingham NG7 2RD, UK; 3 State Key Laboratory of Silicate Materials for Architectures, Wuhan University of Technology, Wuhan 430070, China; liuqt@whut.edu.cn; 4 Xinjiang Urban Construction (Group) Co., Ltd., Wulumuqi 830000, China; xjgongrf@163.com

**Keywords:** low-temperature construction additive, preparation method, volumetric properties, modification mechanism, mixture performance

## Abstract

Green production of asphalt materials is very important to promote energy savings and emission reduction during the construction and maintenance of asphalt pavement. A low-temperature construction additive (LCA) made from the waste plastic and waste rubber is proposed, which belongs to a class of environmentally friendly additives for asphalt mixtures. Marshall stability was tested to evaluate the mechanical performance of LCA-modified asphalt mixtures (LCA-AMs). In order to determine the best preparation parameters of LCA-AMs, the influence of the content and LCA addition method on the strength of LCA-AMs was studied. In addition, the impact of epoxy resin (ER) on the mixtures’ performances was evaluated. The results show that the LCA can significantly reduce the formation temperature of asphalt mixtures, and the resulting asphalt mixtures have good workability in a lower temperature range (90–110 °C). The ER should be added to the LCA-AMs after 4 h of curing. All the volumetric properties satisfy the technical requirements. The low-temperature crack resistance and fatigue resistance of LCA-AMs were obviously improved with appropriate dosages of ER, which can effectively improve the mechanical performance of the asphalt mixtures. The ER can significantly increase the rutting resistance and water sensitivity of LCA-AMs, therefore making it feasible to improve the mixture performance by the enhancement provided by a low dosage of ER.

## 1. Introduction

Asphalt pavement is a kind of continuous pavement without joints, which is the main form of highway pavement and has many advantages, such as smoothness, low noise, durability, anti-skid properties and easy maintenance [1]. The mixing temperature during the production process of hot-mixed asphalt mixtures (HMAs) is around 150–180 °C [2,3], and these need to be kept at A high temperature during the production, transportation and paving stages [4]. However, heating the asphalt mixture to high temperature requires a lot of fuel consumption [5], and emits greenhouse gases and toxic gases [6]. Besides this, the high construction temperature of HMA limits its application in cold areas [7]. The concepts of saving energy and consumption reduction have promoted the green production of asphalt materials, which has made researchers pay attention to the warm mix asphalt (WMA) [3,8].

WMA technology originated in Europe [9], and has been widely used all over the world [10]. The purpose of WMA technology is to reduce the viscosity of asphalt binder at high temperature [11,12] by various methods such as the use of organic viscosity reduction additives [13], the foaming viscosity reduction method [14] or using surface-active viscosity reduction additives [15] without reducing the construction quality and mixture performance of the resulting asphalt mixtures, so as to reduce the mixing temperature of these mixtures [8,16]. Compared with HMA, WMA can reduce fuel consumption by more than 40% and reduce the asphalt aging effects caused by high temperatures [17]. Liseane [18] studied the emission and energy consumption of different kinds of asphalt mixtures, and found that the formation temperature of WMA was 20–50 °C lower than that of HMA, and the greenhouse gas emissions were decreased by more than 40%. Maríadel [19] found that the reduction of the mixing temperature can effectively reduce the harmful gases such as CO_2_ and SO_2_ produced during the asphalt mixture production process. Mosa [20] used alum instead of traditional WMA additives. The results showed that alum could reduce the mixing temperature and compaction temperature by 25.5 °C and 20 °C, respectively. Gao [21] indicated that a small amount of WMA additive can reduce the viscosity of asphalt by more than 80%. Huang [22] and others studied the fatigue and mechanical properties of WMA by the discrete element model and found that the fatigue and mechanical properties of warm-mixed rubber asphalt mixture are better than those of ordinary asphalt mixtures. Although WMA is widely used and is considered a mature technology, studies have pointed out that its performance is actually rather poor. For example, Kim [23] studied the properties of WMA and HMA with the same performance graded (PG) asphalt binder and aggregate gradation, and the results showed that WMA had poorer rutting resistance than HMA. Tan [24] used the Marshall design method to study the physical, mechanical properties and mixture performance of WMA. The results showed that the optimum asphalt content of WMA is 0.1–0.2% higher than that of HMA, the air voids and flow value of WMA were slightly higher than those of HMA, and that the immersion residue Marshall strength of WMA is increased, but the freeze-thaw indirect tensile strength decreases.

Low-temperature construction additives (LCAs) are a new environmentally friendly asphalt modifier made from waste plastics or waste rubber [25]. The mixing temperature of LCA-modified asphalt mixtures (LCA-AMs) is even lower than that of WMA, but higher than the mixing temperature of cold mix asphalt mixture (CMA) [26]. Like WMA, LCAs can solve the technical problems of serious pollution and excessive energy consumption of HMA. LCA-AMs can provide a better viscosity reduction effect, and the preparation temperature of asphalt mixtures can be decreased by more than 40 °C, therefore the mixtures have better workability at lower temperatures (90–110 °C), and energy consumption and harmful gas emissions are reduced. They can be directly used for repairing and maintaining the surface layer of new pavement or old pavement [27]. LCA- modified asphalt binders have good storage stability, and it can be used at any time. Due to the small size of the LCA molecules, they may help improve the low-temperature performance and fatigue performance of mixtures [28]. Although LCA-AMs have many advantages such as greater workability, the initial mechanical properties of LCA-AMs are considered insufficient because LCAs reduce the viscosity of asphalt binders [29,30].

In this paper, LCA-AMs were designed and prepared. Firstly, the mechanical properties and preparation parameters of asphalt mixtures with and without LCAs were studied by the Marshall method. The influence of mixing methods and dosage of LCAs on the strength of LCA-AMs were investigated, so as to determine the best LCA-AM preparation parameters. Then a low-dose of epoxy resin (ER) (Hunan Baxiongdi New Material CO., LTD., Changsha, China) was applied to improve the initial mechanical properties of the LCA-AMs. Finally, mixture performance tests were conducted to study the rutting resistance, low-temperature crack resistance, water sensitivity and fatigue resistance of the thus prepared LCA-AMs. 

## 2. Materials and Experimental Methods

### 2.1. Materials

#### 2.1.1. Asphalt Binder

The original asphalt binder was 70# base asphalt binder (Hubei Guochuang Hi-tech Materials CO., LTD., Wuhan, China). The technical performance of this asphalt binder is shown in Table 1. 

#### 2.1.2. LCAs

**Figure 1:**
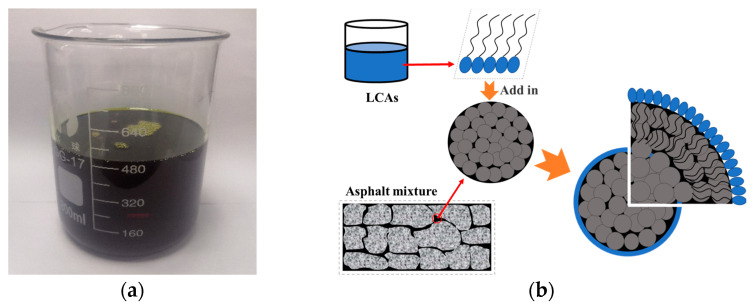
LCAs and their modification process in asphalt binder. (**a**) LCAs; (**b**) LCAs’ mechanism of action.

LCAs are new environmentally friendly additives for asphalt mixtures, made from waste plastics and waste rubber. Among them, benzyl ethylene block copolymer accounts for about 80%, while ER and other additives account for about 20%. Figure 1a shows a typical LCA. After mixing with the asphalt binder, molecules containing amide groups are formed by ionization and recombination, and can form a dense film on the surface of asphalt molecules. LCAs can reduce the surface free energy and surface tension of asphalt molecules, resulting in a decrease in viscosity. At the same time, the lipophilic groups such as higher aliphatic chains, phenolic groups and benzene ring groups in ER can be adsorbed by asphalt molecules, which improves the stability of their dispersion system [31,32,33]. The mechanism of action of LCAs is shown in Figure 1b.

#### 2.1.3. LCAs Modified Asphalt Binder

The LCA-modified asphalt binder was made by the melt blending method. First the 70# asphalt binder was heated to 110 °C. Then, 9% LCA was added to the 70# asphalt binder and shear mixed for 30 min. The optimum 9% dosage of LCA was determined in our previous research [27]. The technical information of the LCA-modified asphalt binder is shown in Table 2.

#### 2.1.4. Aggregates and Filler

The aggregates were divided into the coarse aggregate (≥2.36 mm) and fine aggregate (0–2.36 mm). The coarse aggregates were basalt, the fine aggregates were limestone, and the filler was limestone powder. The technical properties of the aggregates and fillers are listed in Table 3, Table 4 and Table 5.

### 2.2. Design of LCA-Modified Asphalt Mixtures (LAC-AMs)

#### 2.2.1. Mix Design of LCA-AMs

**Figure 2:**
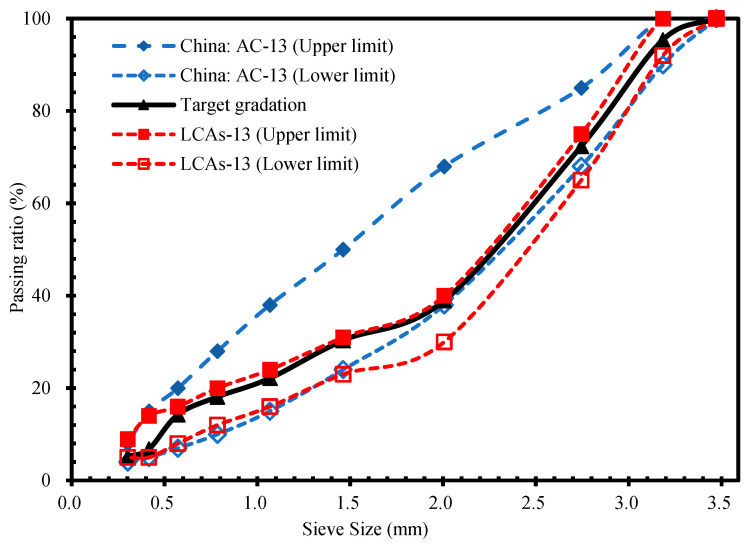
Composite aggregate gradation of asphalt mixture.

A skeleton dense-graded mixture was used in this research, where the nominal maximum aggregate size of the mixture was 13 mm (LCAs-13). The aggregates were divided into four grades: 10–15 mm, 5–10 mm, 3–5 mm and 0–3 mm. The composite aggregate gradation range of LCAs is shown in Figure 2, and it has some common areas with the Chinese dense-graded mixture (AC-13). Both AC-13 and LCAs-13 are commonly used mixtures for the surface layer of asphalt pavement. The target gradation of LCAs-13 is shown in Figure 2, and the mixing ratio of aggregates is shown in Table 6. The asphalt content was 4.8% by weight of aggregate.

#### 2.2.2. Manufacturing Temperature of LCA-AMs

The heating temperature of conventional HMA is clearly specified in JTG F40-2004. The heating and mixing temperatures for LCA-AMs are shown in Table 7. As it can be seen from Table 7, compared with the conventional HMA, the heating temperature of asphalt binder is reduced by 40 °C, the heating temperature of aggregate is reduced by 50 °C, and the mixing temperature of LCA-AMs is reduced by 30 °C.

#### 2.2.3. Preparation Method of LAC-AMs

**Figure 3:**
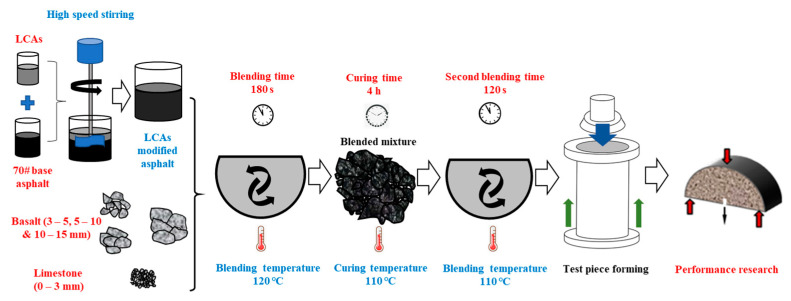
Preparation method of LAC-AMs.

The aggregates, filler and LCA-modified asphalt binder were mixed at 120 °C for 180 s. The prepared LAC-AM was cured at 110 °C for 4 h. The high temperature curing could accelerate the volatility of volatile components in the LCA, increase the viscosity of LCA- modified asphalt binder, and promote the development of strength in the LAC-AM. The cured LAC-AM was mixed again at 110 °C for 120 s to prepare specimens, which were then stored at room temperature (25 °C) for 72 h before further tests. The preparation method is shown in Figure 3.

### 2.3. Experimental Methods

#### 2.3.1. Volumetric Properties and Mechanical Performance Tests

The specimens were compacted using the Marshall method at 110 °C with both sides of specimens compacted 100 times, the specimens were then cured for 72 h at room temperature (25 °C). The volumetric properties and mechanical properties of specimens, including the volume of air voids (VV), the voids in mineral aggregate (VMA), the voids filled with asphalt (VFA), the Marshall stability (MS) and flow value (FL), were tested based on JTG E20-2011.

#### 2.3.2. ER Dosage and Mixing Methods

**Figure 4:**
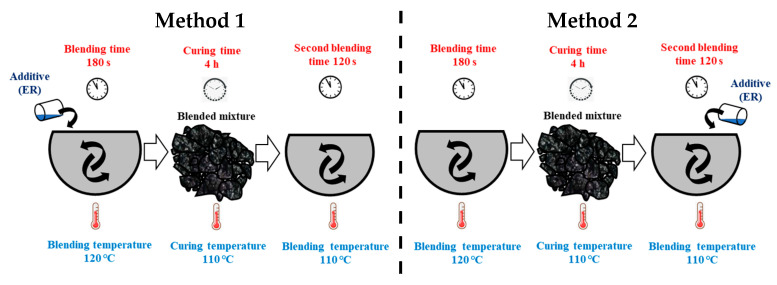
Different ER mixing methods.

Epoxy resin (ER) at different dosages (0.3%, 0.6%, 1.0%, 2.0% and 4.0%) and different mixing methods were applied to study the differences between adding ER to LCA-AMs before and after curing, as shown in Figure 4. Method 1 involved adding ER before curing, while in Method 2 ER was added after curing for 4 h at 110 °C. The MS of specimens was tested after curing 72 h at room temperature (25 °C).

#### 2.3.3. Rutting Resistance Test

The slabs used for the rutting resistance tests were prepared using the wheel tracking method (JTG E20 T0703). The rutting resistance tests were conducted according to the criteria of JTG E20 T0910. The rutting resistance was tested by the wheel track test machine (Beijing Aerospace Keyu Test Instrument, Beijing, China) at a wheel rolling load of 0.7 MPa and a rate of 42 times/min. The test temperature was 60 °C. After curing at 25 °C for 72 h, specimens (300 mm × 300 mm × 50 mm) were put in the wheel track machine for 5 h at 60 °C. The dynamic stability (DS) of LCAs-AM was then calculated according to Equation (1).

(1) $$\mathrm{DS}=\frac{\left(t_{2}-t_{1}\right)\times N}{\left(d_{2}-d_{1}\right)}$$

where DS is the dynamic stability of asphalt mixture (times/mm), *t*_1_ and *t*_2_ is the 45 min and 60 min, respectively, *d*_1_ and *d*_2_ are the rutting depths of asphalt mixtures at *t*_1_ and *t*_2_ respectively (mm), *N* is the speed of the testing wheel (42 times/min).

#### 2.3.4. Low-Temperature Resistance Test

**Figure 5:**
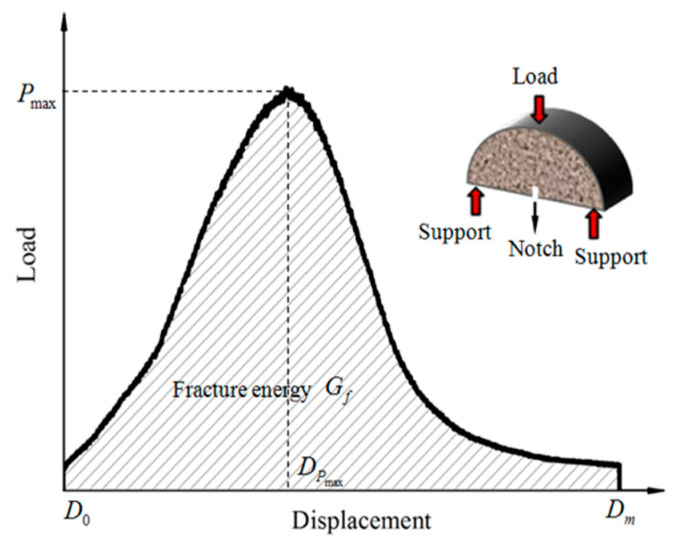
Load-displacement curve and test setup of SCB tests.

The low-temperature crack resistance was tested by semi-circular bending (SCB) tests at the loading rate of 1.27 mm/min using a Universal Testing Machine (UTM-100); the distance between two supports was set to 75 mm. The specimen (φ 101.6 mm × 63.5 mm specimen) was cut into two semicircles, and a notch (5 mm length × 3 mm width) was cut along the height direction from the midpoint of the semicircle, as shown in Figure 5. The specimens were put into the UTM at −10 °C and 0 °C for 4 h before testing.

#### 2.3.5. Water Sensitivity Test

The immersion Marshall test and freeze–thaw split test were used to study the water sensitivity. The specimens for both tests (φ 101.6 mm × 63.5 mm) were tested at loading rates of 50 mm/min. Both sides of the specimens were compacted 100 times and 75 times, for the immersion Marshall test and freeze–thaw split test, respectively. The immersion Marshall test was conducted according to the criteria of JTG E20 T0709. The samples of unconditional group and conditional group were immersed in the 60 °C water bath for 30 min and 48 h respectively. The freeze–thaw split test was conducted according to the criteris of JTG E20 T0729. The samples of unconditional group were immersed in the 25 °C water bath for 2 h; for the conditional group, the Marshall specimens were first vacuum-filled with water, and the vacuum degree was 97.3–98.7 kPa, then frozen at −18 °C for 16 h. after that, put the specimens in 60 °C water bath for 24 h, and finally put in a 25 °C water bath for 2 h.

#### 2.3.6. Fatigue Resistance Test

The fatigue resistance was tested by the recycle semi-circular bending (R-SCB) test under different stress ratios of 0.3, 0.4, 0.5, 0.6 and 0.7 at the loading frequency of 2 Hz using UTM-100. The specimens were conditioned at 25 °C for 4 h, and the distance between supports was set to 75 mm.

## 3. Results and Discussions

### 3.1. Volumetric Properties of LCAs-AM

The volumetric properties and mechanical performance of LCAs-AM, including the volume of air voids (VV), the voids in mineral aggregate (VMA), the voids filled with asphalt (VFA), the Marshall stability (MS) and flow value (FL), are shown in Table 8. The comparison of volumetric properties and mechanical performance between 70# asphalt mixture (70#-AM) and LCA-AM is shown in Table 9. From Table 8, all of the volumetric properties meet the design requirements. From Table 9, compared to the 70#-AM, the VFA and MS of LCA-AM decreased by 1.5% and 28.3%, respectively, while the VV, VMA and FL of the LCA-AM increased by 7.5%, 4.0% and 25.7%, respectively. This indicates that adding LCAs decreases the viscosity of the asphalt mixture, resulting in the decrease of its MS.

The LCAs in asphalt binder produce amide-containing molecules by ionised recombination, forming a dense film on the surface of the molecules. With the increase of temperature, the surface energy of the molecules decreases and subsequently their surface tension decreases, leading to a decrease in viscosity. As the viscosity decreases, the cohesion of the mixture is reduced, leading to a decrease in MS and an increase in FL. Although the average MS of 7.56 kN is slightly below the specification requirement of 8 kN, the LCA-AM strength will improve as the volatile solvents in the LCA gradually evaporate. In addition, the LCA contains around 20% ER, which can be used to improve the initial mechanical performance of LCA-AMs by adding a small amount of epoxy additive.

### 3.2. Factors Affecting the Mechanical Performance of LACs-AM

#### 3.2.1. Mixing Methods

Three dosages of ER (1.0%, 2.0% and 4.0%) and two mixing methods (Method 1 and Method 2) were applied to investigate the effect of different ER dosages and mixing methods on the mechanical performance of LCA-AMs, the results of which are shown in Table 10. From Table 10, using Method 1 slightly improves the mechanical performance of the LCA-AM, with 1.0%, 2.0% and 4.0% EP increasing the MS by 2.51%, 6.22% and 15.34%, respectively. Using Method 2 greatly improves the MS of LCAs-AM, by 80.56%, 92.86% and 98.02% for 1.0%, 2.0% and 4.0% EP, respectively. The improvement of 1.0% EP on the MS is more than 30 times greater with Method 2 than with Method 1. Therefore, the mechanical performance of LCAs-AM is much better using Method 2 than Method 1.

By using Method 1, the curing reaction is already finished during the 110 °C curing process, and it cannot further enhance the mechanical performance during the secondary mixing, compaction and curing processes of the LCA-AM. Therefore, the ER effect on improving the mechanical performance is greatly decreased for Method 1. However, by using Method 2, the curing reaction and strength formation of ER happen after the manufacturing of the LCA-AM specimens, so it can produce an obvious reinforcing effect on the mechanical performance of LCA-AMs.

#### 3.2.2. ER Dosage

**Figure 6:**
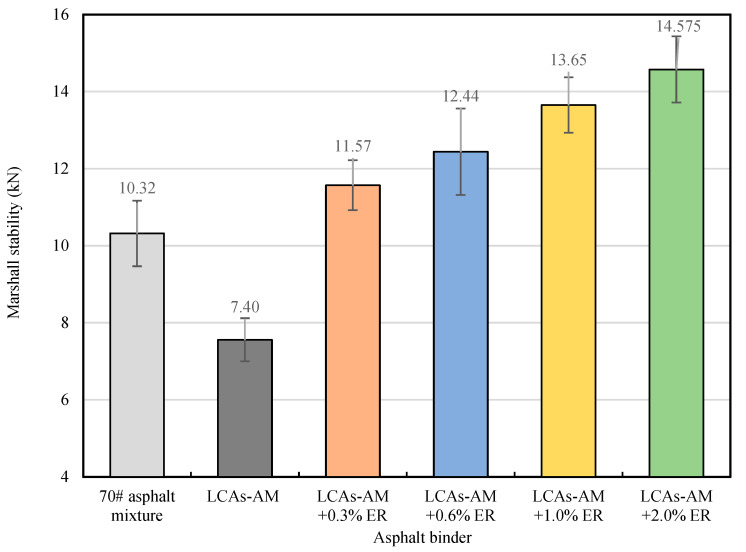
MS of LCA-AMs with different dosages of ER.

Four different dosages of ER (0.3%, 0.6%, 1.0% and 2.0%) are applied to LAC-AMs and compared to LAC-AM without ER. The mechanical performance results are shown in Figure 6. It can be seen that the MS of 0.3%, 0.6%, 1.0% and 2.0% ER-reinforced LCA-AMs are 12.11%, 20.54%, 32.27% and 41.28% higher than those of the original AM, and 56.35%, 68.11%, 84.46% and 97.03% higher than that of the LCA-AM without ER. With the addition of only 0.3% ER, the mechanical performance of the ER-reinforced LCA-AM is more than 50% higher than that of the LCA-AM without ER, which shows that the addition of a small amount of ER can effectively improve the mechanical performance of LCA-AMs.

### 3.3. Mixture Performance of LCA-AMs

#### 3.3.1. Rutting Resistance

**Figure 7:**
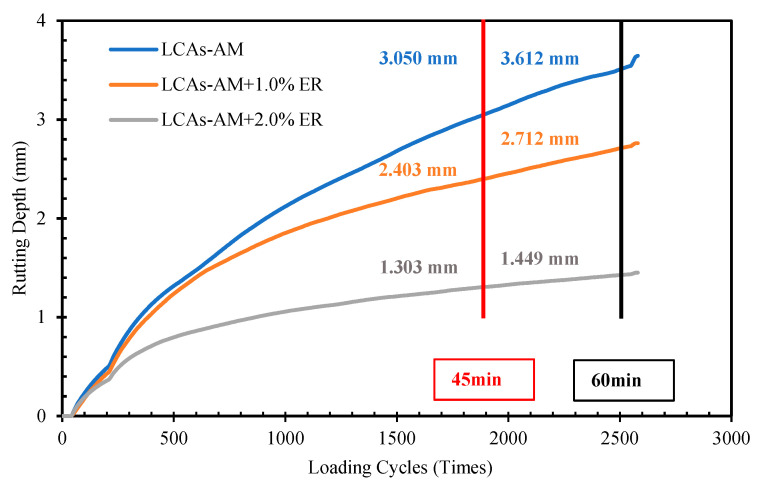
Rutting depth increase curve of asphalt mixtures.

**Figure 8:**
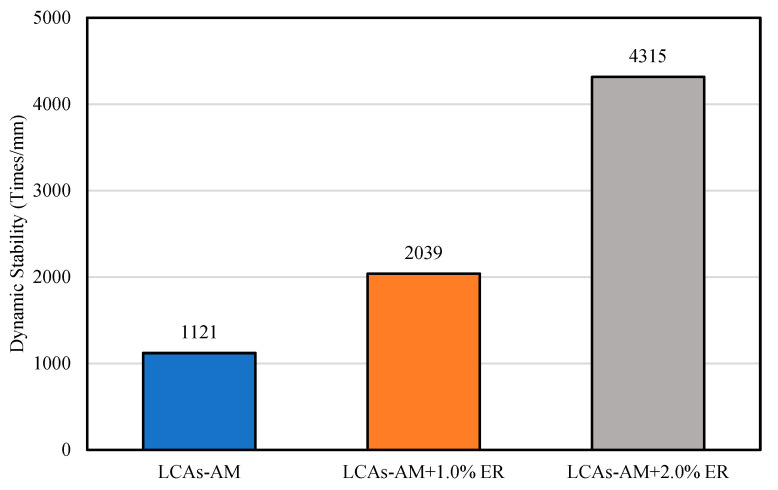
Dynamic stability of asphalt mixtures.

The wheel track test is used to study the rutting resistance of 1.0% and 2.0% ER-100 reinforced LCA-AMs and the original asphalt mixture. The specimens were cured at 25 °C for 72 h. The results are shown in Figure 7 and Figure 8, where it can be seen that the accumulated deformation (rutting depth) of the asphalt mixture increases gradually with the increase of the number of load cycles, and the rutting depth growth rate of ER-100-reinforced LCA-AM is less than that of the LCA-AM without ER. After the same number of load cycles, the rut depth of ER-reinforced LCA-AMd is much smaller than that of the LCA-AM without ER. At the loading times of 45 min and 60 min, the rut depth of the LCA-AM with 1.0% ER is 21.2% or 25.5% smaller than that of the original asphalt mixture; the rutting depth of 2.0% ER-reinforced LCA-AM is 57.3% (59.9% smaller than that of the LCA-AM without ER). Meanwhile, the dynamic stability of ER-reinforced LCA-AMs is much higher than that of the LCA-AM without ER. The results indicate that ER can significantly enhance the high-temperature stability of LCA-AMs. The reason is that, after the curing of the ER, part of the strength of the asphalt mixture is provided by the ER, and the ER can form a spatial skeleton structure in the asphalt mixtures, which have greater strength and stiffness. Therefore, it can greatly improve the rutting resistance of the mixtures.

#### 3.3.2. Low-Temperature Crack Resistance

**Figure 9:**
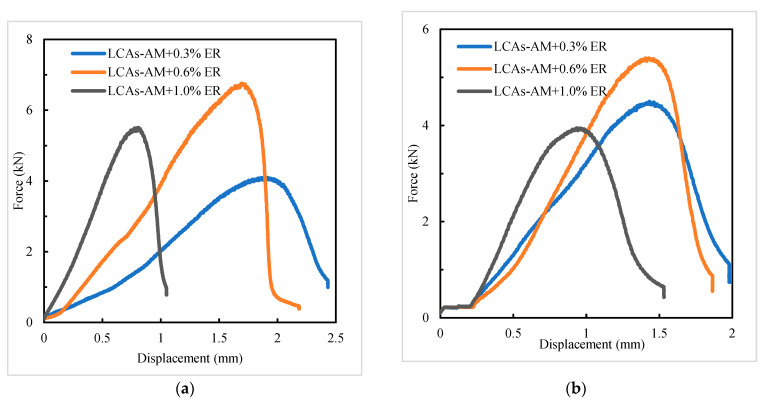
Displacement and load curve of SCB tests: (**a**) −10 °C; (**b**) 0 °C.

**Figure 10:**
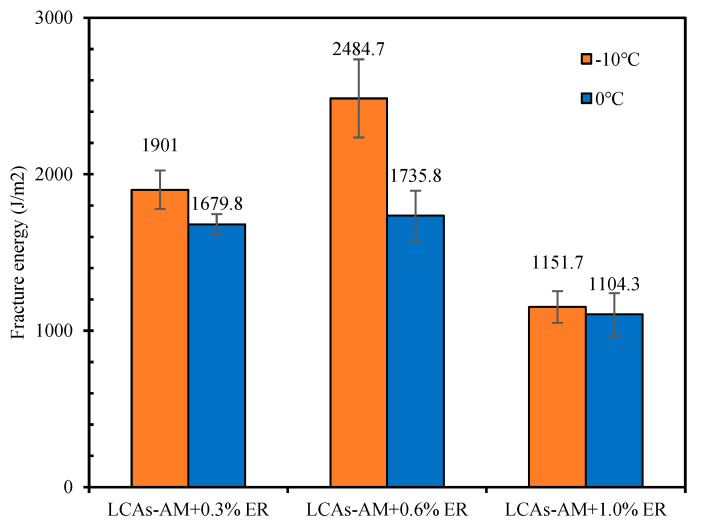
Low-temperature fracture energy with different ER contents.

LCAs can decrease the formation temperature of asphalt mixtures by decreasing the viscosity of asphalt binders. However, if the reduction in viscosity is too large, it results in a reduction in the mechanical properties of the asphalt mixture (even at low temperature), which can easily break down during low-temperature crack resistance tests. In order to improve the low-temperature crack resistance of LCA-AMs, −10 °C and 0 °C SCB tests were conducted to evaluate the low-temperature cracking resistance of LCA-AMs with different dosages of ER. The results are shown in Figure 9 and Table 11, and the comparison of fracture energy of LCAs-AM is shown in Figure 10. According to Figure 9 and Table 11, the fracture energy and fracture toughness of LCA-AMs increase first and then decrease with the increase of ER dosages, and the LCA-AM with 0.6% ER has the highest fracture energy and fracture toughness. After curing, the ER acts as part of the skeleton structure in LCA-AMs. Therefore, a low content of ER can enhance the significantly increase the low-temperature crack resistance of LCA-AMs.

However, with the gradual increase of ER dosages, the increasing effect of the ER on the stiffness (mechanical performance) is much more obvious, and it may be too big and have a negative effect on the low-temperature crack resistance of LCA-AMs. The ER content in the LCAs-AM does not need to be high. The results show that one can significantly increase the low-temperature crack resistance of LCA-AMs by controlling the ER content in LCA-AMs.

#### 3.3.3. Water Sensitivity

##### Immersion Marshall Test

**Figure 11:**
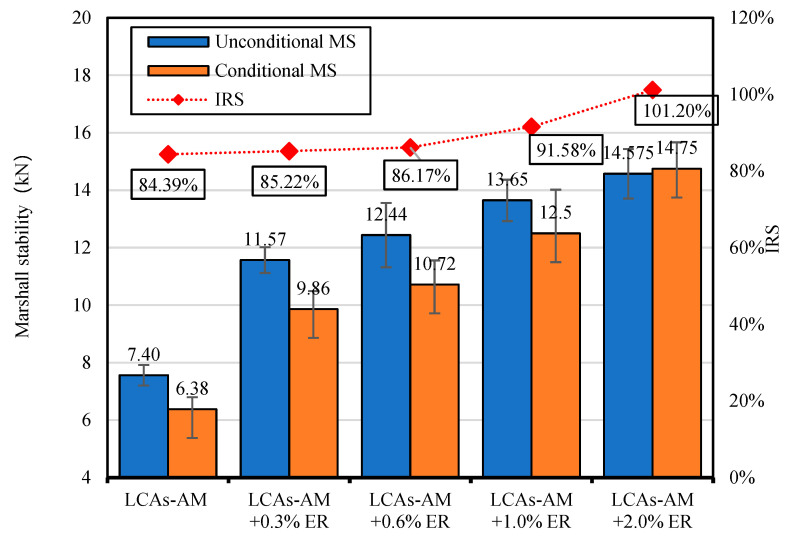
Immersion Marshall stability and RMS of LCA-AMs with ER.

The residual Marshall stability (RMS) of LCA-AMs with different dosages of ER were studied using the immersing Marshall test. The results are shown in Figure 11. From Figure 11, the Marshall stability of LCA-AMs with ER is higher than 8 kN both before and after the immersion condition. The RMS of LCA-AMs increases with the increase of ER dosage, and all the RMS of LCA-AMs are more than 80%. The results even satisfy the requirements of the specification of HMA.

##### Freeze-Thaw Splitting Test

**Figure 12:**
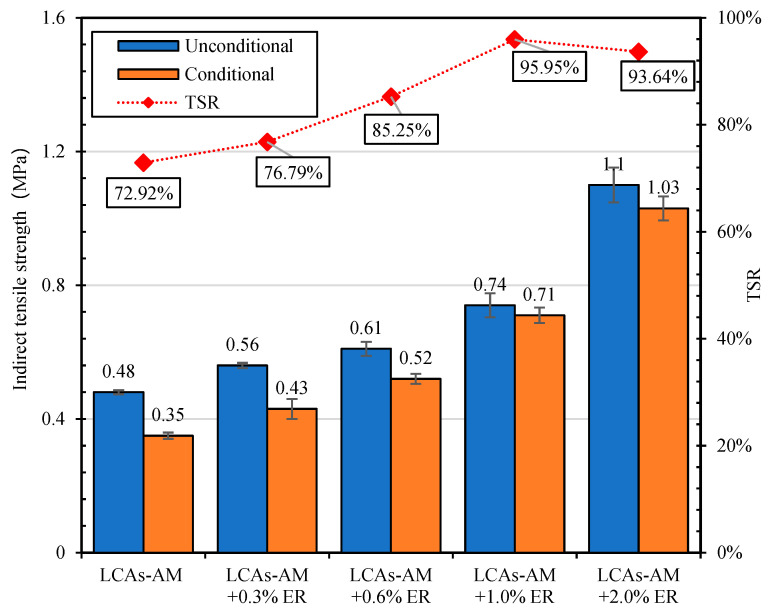
Indirect tensile strength and TSR of LCA-AMd with ER.

The freeze-thaw splitting ratio (TSR) of LCA-AMs with the different dosages of ER were studied using the freeze-thaw splitting test. The results are shown in Figure 12. It can be seen from Figure 12 that the TSR of LCA-AM without ER is lower than 75%. However, all the TSRs of LCA-AMs with ER are higher than 75%, and meet the requirements of HMA. The TSR increases first and then decreases with the increase of ER dosage, and the TSR of LCA-AMs with 1% ER is the highest. Therefore, due to the enhancement effect of ER, LCA-AMs have a good anti-water damage performance.

#### 3.3.4. Fatigue Resistance

**Figure 13:**
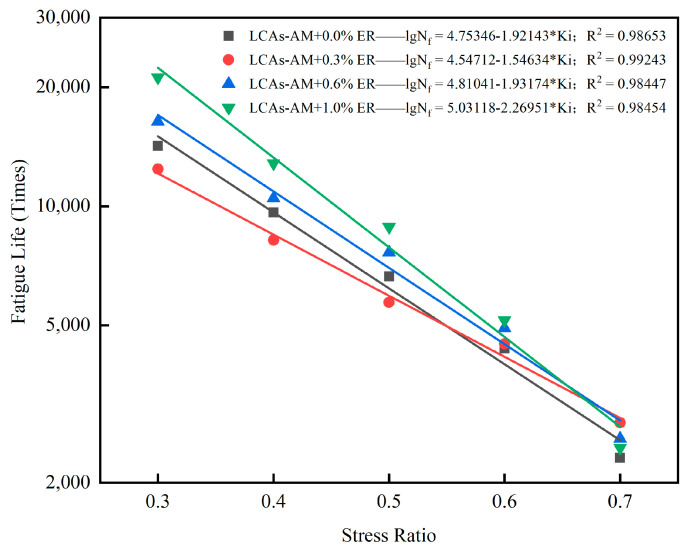
Fatigue life of LCA-AMs with ER.

The repeated SCB test was conducted to investigate the fatigue resistance of LCA-AMs. The results are shown in Table 12 and Figure 13. The stress ratios of the fatigue test were 0.3, 0.4, 0.5, 0.6 and 0.7, respectively.

It can be seen from Table 12 that, at 25 °C, the peak load and fracture toughness of LCA-AM with 1.0% ER are the highest, while the LCA-AM with 0.6% ER has the maximum fracture work and fracture energy. Compared with the LCA-AM without ER, the fracture energy of LCA-AMs with 0.3%, 0.6% and 1.0% ER increase by 26.7%, 109.3% and 60.5%, respectively. It can be seen from Figure 13 that under the same loading frequency, the fatigue life decreases gradually with the increase of stress ratio. When the stress ratio increases from 0.3 to 0.7, the fatigue life of the LCA-AM without ER decreases by 83.7%, which shows that the fatigue resistance of the asphalt mixture has an obvious stress dependence. When the stress ratio is 0.3, the fatigue life of the LCA-AM increases gradually with the increase of ER content. Compared with the LCA-AM without ER, the fatigue performance of LCA-AM with 0.3% ER decreased by 12.5%, while that of LCA-AMs with 0.6% and 1.0% ER increase by 15.3% and 48.9%, respectively. When the stress ratio is 0.7, the fatigue resistance of 0.3%, 0.6% and 1.0% ER reinforced LCA-AMs increases by 22.6%, 11.8% and 6.0%, respectively. This shows that ER can significantly increase the fatigue resistance of LCA-AMs. The LCAs-AM with 0.6% and 1.0% ER are much better than the LCA-AM without ER.

## 4. Conclusions

In this paper, an environmentally friendly LCA asphalt modifier additive was developed, which can used to decrease the construction temperature of asphalt mixtures. The design and preparation parameters of LCA-modified asphalt mixtures were determined. The volumetric properties, mechanical performance and mixture performance of LCA-AMs were studied as well. The main conclusions are as follows:

(1) LCAs can significantly decrease the preparation temperature of asphalt mixtures. All the volumetric properties, namely the VV, VMA, VFA, MS and FL of LCA-AMs meet the design requirements, however, the MS of original LCA-AM (7.56 kN) is slightly lower than 8 kN.
(2) The content and mixing method (Method 1 and Method 2) of ER have obvious effects on the mechanical performance of LCA-AMs. For instance, 0.3% ER can effectively enhance the mechanical properties of LCA-asphalt mixtures, and the MS is 56.4% higher than that of LCA-AM without ER. The MS of 1% ER-reinforced LCA-AMs mixed by Method 2 is 30 times higher than that of LCA-AMs mixed by Method 1.
(3) The ER can form a spatial skeleton structure in the mixture, and effectively improve the initial strength of the asphalt mixture. The enhancement effect of ER can significantly increase the rutting resistance and water sensitivity of LCA-AMs.
(4) 0.3% and 0.6% ER dosages can improve the fracture energy and fracture toughness of LCA-AMs, and 0.6% and 1.0% ER can increase the fatigue life of LCA-AMs. The low-temperature crack resistance and fatigue resistance of LCA-AMs with appropriate dosages of ER will be obviously improved compared with the LCA-AM without ER.

It is feasible to improve the mixture performance by the enhancement of low dosages of ER. LCAs can facilitate the low-temperature preparation of asphalt mixtures, with remarkable environmental benefits. LCAs should find wide application prospects in asphalt pavement engineering.

## Figures and Tables

**Table 1:** Technical information of the 70# asphalt binder [27].

Status	Technical Information	Units	Results	Requirement	Methods
Before TFOT	Penetration (100 g, 5 s, 25 °C)	0.1 mm	71	60–80	ASTM D5
	Ductility (10 °C)	cm	42.0	≥25	ASTM D113
	Softening point	°C	47.9	≥46	ASTM D36
After TFOT	Penetration (100 g, 5 s, 25 °C)	0.1 mm	63	≥61	ASTM D5
	Ductility (10 °C)	cm	14.3	≥6	ASTM D113
	Softening point	°C	51.0	–	ASTM D36

**Table 2:** Technical information of the LCA-modified asphalt binder.

Technical Information		Units	Results	Requirement	Methods
Brookfield rotational viscosity (100 °C)		Pa·s	1.2	≤1.5	JTG E20 T0625
Penetration (100 g, 5 s, 25 °C)		0.1 mm	174	–	ASTM D5
Ductility (10 °C)		cm	≥150	≥100	ASTM D113
Softening point		°C	38.2	–	ASTM D36
Flash point		°C	175	≥160	JTG E20 T0611
After TFOT	Mass change	%	−0.5	−5–+5	JTG E20 T0610
	Penetration	0.1 mm	168	Measure	ASTM D5
	Ductility (10 °C)	cm	≥150	≥100	ASTM D113
	Softening point	°C	41.3	≥40	ASTM D36

**Table 3:** Technical properties of coarse aggregate.

Parameters	Unit	Results	Requirements	Experimental Method
Stone crushing value	%	13	≤28	JTG E42 T0316
Needle flake content	%	7.6	≤15	JTG E42 T0312
Los Angeles wear value	%	16	≤28	JTG E42 T0317
Water absorption	%	0.9	≤2.0	JTG E42 T0308
Apparent density	–	2.838	≥2.6	JTG E42 T0605

**Table 4:** Technical properties of fine aggregate.

Parameters	Unit	Results	Requirements	Experimental Method
Sediment percentage	%	2.4	≤3	JTG E42 T0335
Sand equivalent	%	72.6	≥60	JTG E42 T0334
Angularity (flow time method)	s	42.7	≥30	JTG E42 T0345
Apparent density	–	2.667	≥2.5	JTG E42 T0328

**Table 5:** Technical properties of mineral powder.

Parameters		Unit	Results	Requirements	Experimental Method
Apparent density		–	2.701	≥2.50	JTG E42 T0352
Particle size range (%)	<0.6	mm	100	100	JTG E42 T0351
	<0.15	mm	97.2	90–100	
	<0.075	mm	90.8	75–100	
Plasticity coefficient		–	3.2	<4	JTG E42 T0354
Hydrophilic coefficient		–	0.6	<1	JTG E42 T0353

**Table 6:** Mixing ratio of aggregates.

Aggregate	10–15 mm	5–10 mm	3–5 mm	0–3 mm	Filler
Mixing ratio (%)	36	6	25	30	3

**Table 7:** Construction temperature of conventional HMA and LCAs-AM.

Asphalt Mixture	Asphalt Heating Temperature (°C)	Aggregate Heating Temperature (°C)	Mixing Temperature (°C)	Secondary Mixing Temperature (°C)
HMA	145	165	150	–
Asphalt mixture with LCA	105	115	120	110

**Table 8:** Volumetric properties and mechanical performance of LCAs-AM.

Specimen Number	Maximum Theoretical Relative Density	VV (%)	VMA (%)	VFA (%)	MS (kN)	FL (mm)
1	2.553	4.3	15.6	72.4	7.34	4.6
2		4.5	15.8	71.5	7.21	4.4
3		4.3	15.6	72.4	6.84	4.5
4		4.3	15.5	72.3	7.82	4.4
5		4.2	15.3	72.5	7.74	4.1
Average value		4.3	15.6	72.2	7.40	4.4

**Table 9:** Comparison of volumetric properties and mechanical performance between 70#-AM and LCAs-AM.

Asphalt Mixture	VV (%)	VMA (%)	VFA (%)	MS (kN)	FL (mm)
70#-AM	4.0	15.0	73.3	10.32	3.5
LCAs-AM	4.3	15.6	72.2	7.40	4.4
Specification requirements	3–5	≥13	65–75	≥8	3–6

**Table 10:** MS of LCA-AMs prepared with different mixing methods.

ER Dosages	MS (kN)	
	Method 1	Method 2
1.0%	7.75	13.65
2.0%	8.03	14.58
4.0%	8.72	14.97

**Table 11:** SCB test results of LCA-AMs.

ER Dosages (%)	Test Temperature (°C)	Peak Load (N)	Fracture Work (J)	Fracture Energy (J/m^2^)	Fracture Toughness (kPa × m^0.5^)
0.3	−10	4112	5.43	1901.0	359.4
	0	4499	4.80	1679.8	392.4
0.6	−10	6774	7.10	2484.7	590.9
	0	5413	4.96	1735.8	472.2
1.0	−10	5518	3.29	1151.7	481.3
	0	3957	3.16	1104.3	345.1

**Table 12:** Results of repeated SCB tests of LCA-AMs.

Asphalt Mixture	ER Dosage (%)	Peak Load (N)	Fracture Work (J)	Fracture Energy (J/m^2^)	Fracture Toughness (kPa × m^0.5^)
LCAs-AM with ER	—	953	0.88	180.6	68.0
	0.3	756	0.65	228.8	65.9
	0.6	1192	1.08	378.0	104.0
	1.0	1268	0.92	289.8	110.5

## Data Availability

The data presented in this study are available on request from the corresponding author.
